# KSHV induces rapid release of angiopoietin-2 from endothelial cells to promote angiogenesis and inflammation

**DOI:** 10.1186/1750-9378-7-S1-P41

**Published:** 2012-04-19

**Authors:** Fengchun Ye, Whitney Greene, Fuchun Zhou, Aaron Weinberg, ShouJiang Gao

**Affiliations:** 1 Department of Biological Sciences, Case Western Reserve University, Cleveland, OH, USA; 2 Department of Pediatrics and Greehey Children’s Cancer Research Institute, UTHSCSA, San Antonio, TX, USA

## 

The development of Kaposi’s sarcoma (KS), the most common malignancy in AIDS patients, results from Kaposi’s sarcoma-associated herpesvirus (KSHV) infection of endothelial cells and subsequent induction of proliferation, angiogenesis, and inflammation. Previously, we demonstrated that KSHV infection of human umbilical vein endothelial cells (HUVEC) induced a transcriptional induction of Angiopoietin-2 (Ang-2), a pro-angiogenic and pro-inflammatory cytokine that is highly present in KS tumors. This transcriptional up-regulation of Ang-2 started at 12 h and peaked at 54 h post-infection. In difference to our previous data, here we demonstrate that KSHV infection of HUVEC induces rapid release of Ang-2 within minutes of viral contact. Pre-made Ang-2 is stored in the Weibel-Palade body in endothelial cells and is released through regulated exocytosis. KSHV binding to HUVEC is responsible for rapid Ang-2 release because blockade of viral binding inhibits this cytokine exocytosis. We find that KSHV binding to its integrin receptors on endothelial cells activates the integrin tyrosine kinase receptors signaling pathways, including tyrosine phosphorylation of the kinases FAK and Src, and triggers rapid calcium mobilization. This mobilization likely plays a key role in mediating Ang-2 release, as its inhibition by various calcium chelators and calcium channel blockers substantially reduces Ang-2 release. We also demonstrate a direct interaction and association of the kinase Src with the alpha1C subunit of L-type calcium channel. Indeed, specific inhibitors of protein tyrosine phosphorylation not only disrupt this interaction but also abolish Ang-2 release. Finally, preliminary data from in vitro cell adhesion assays suggest that this rapidly released Ang-2 enhances migration and adhesion of monocytes to the infected endothelial cells. To our knowledge, this is the first demonstration of interaction between KSHV and its integrins receptors in regulating rapid cytokine release. This study also uncovers a novel mechanism of KSHV induction of angiogenesis and inflammation, which is much faster, and could likely play important roles in the early event of KS tumor development.

